# Diastereomeric Recognition of 5’,8-cyclo-2’-Deoxyadenosine Lesions by Human Poly(ADP-ribose) Polymerase 1 in a Biomimetic Model

**DOI:** 10.3390/cells8020116

**Published:** 2019-02-02

**Authors:** Annalisa Masi, Arianna Sabbia, Carla Ferreri, Francesco Manoli, Yanhao Lai, Eduardo Laverde, Yuan Liu, Marios G. Krokidis, Chryssostomos Chatgilialoglu, Maria Rosaria Faraone Mennella

**Affiliations:** 1 Istituto per la Sintesi Organica e la Fotoreattività, Consiglio Nazionale delle Ricerche, 40129 Bologna, Italy; francesco.manoli@isof.cnr.it (F.M.); chrys@isof.cnr.it (C.C.); 2 Dipartimento di Biologia, Università di Napoli “Federico II”, 80138 Napoli, Italy; ar.sabbia@studenti.unina.it; 3 Department of Chemistry and Biochemistry, Florida International University, Miami, FL 33199, USA; yalai@fiu.edu (Y.L.); yualiu@fiu.edu (Y.L.); 4 Biochemistry Ph.D. Program, Florida International University, Miami, FL 33199, USA; elave006@fiu.edu; 5 Biomolecular Sciences Institute, Florida International University, Miami, FL 33199, USA; 6 Institute of Nanoscience and Nanotechnology, NCSR Demokritos Agia Paraskevi, 15310 Athens, Greece; m.krokidis@inn.demokritos.gr

**Keywords:** human poly(ADP-ribose) polymerase 1 (PARP1), PARP-DNA complex, DNA-protein binding, DNA repair, 5’,8-Cyclopurine-2’-deoxynucleoside, DNA damage, DNA repair efficiency

## Abstract

5’,8-Cyclo-2’-deoxyadenosine (cdA), in the 5’*R* and 5’*S*diastereomeric forms, are typical non strand-break oxidative DNA lesions, induced by hydroxyl radicals, with emerging importance as a molecular marker. These lesions are exclusively repaired by the nucleotide excision repair (NER) mechanism with a low efficiency, thus readily accumulating in the genome. Poly(ADP-ribose) polymerase1 (PARP1) acts as an early responder to DNA damage and plays a key role as a nick sensor in the maintenance of the integrity of the genome by recognizing nicked DNA. So far, it was unknown whether the two diastereomeric cdA lesions could induce specific PARP1 binding. Here, we provide the first evidence of PARP1 to selectively recognize the diastereomeric lesions of 5’*S*-cdA and 5’*R*-cdA in vitro as compared to deoxyadenosine in model DNA substrates (23-mers) by using circular dichroism, fluorescence spectroscopy, immunoblotting analysis, and gel mobility shift assay. Several features of the recognition of the damaged and undamaged oligonucleotides by PARP1 were characterized. Remarkably, PARP1 exhibits different affinities in binding to a double strand (ds) oligonucleotide, which incorporates cdA lesions in *R* and *S* diastereomeric form. In particular, PARP1 proved to bind oligonucleotides, including a 5’*S*-cdA, with a higher affinity constant for the 5’*S* lesion in a model of ds DNA than 5’*R*-cdA, showing different recognition patterns, also compared with undamaged dA. This new finding highlights the ability of PARP1 to recognize and differentiate the distorted DNA backbone in a biomimetic system caused by different diastereomeric forms of a cdA lesion.

## 1. Introduction

Purine 5’,8-cyclo-2’-deoxynucleosides (cdPu), i.e., 5’,8-cyclo-2’-deoxyadenosine (cdA) and 5’,8-cyclo-2’-deoxyguanosine (cdG), are typical oxidized DNA lesions induced by an hydroxyl radical (HO^•^) abstraction of the H5’ atom of the 2-deoxyribose moiety, followed by radical cyclization and oxidation steps, which creates a new covalent bond, locking the sugar and purine moieties [1,2].

**Figure 1:**
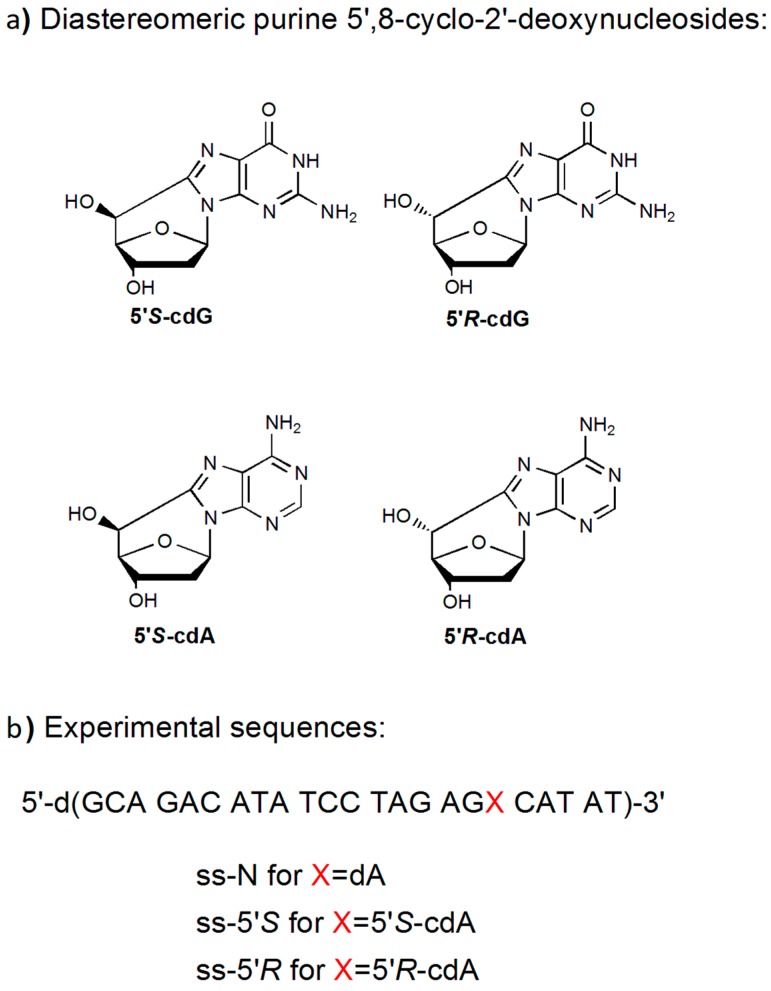
Structures and oligonucleotide sequences. (**a**) Chemical structures of the diastereomeric purine 5’,8-cyclo-2’-deoxynucleosides. (**b**) The sequences of the single stranded (ss) oligonucleotides used in this study. In the double stranded (ds) oligonucleotides, the template strand contains a ds-N (dA), or ds-5’*S* or ds-5’*R* and the complementary strand contains a T opposite to X.

These lesions can exist in the two diastereomeric forms, 5’*R* and 5’*S* (Figure 1a) [1,2]. They are highly mutagenic in vitro and in vivo [3,4] and their accumulation in cellular DNA is associated with aging, cancer, and neurodegeneration [5,6,7,8,9].

Numerous studies have shown that the concentrations of O_2_, higher than physiological conditions, can inhibit the formation of cdPu lesions by reacting with the C5’ radical, thereby preventing the cyclization reaction [10,11,12]. As the detection and quantification of these lesions are concerned, they do not suffer from oxidative artifacts during work-up, like the well-known 8-oxo-dG [2,12]. The significant resistance of the glycosidic bond of cdPu to hydrolysis along with the proven oxygen stability make these lesions a robust biomarker of oxidative DNA damage, in particular that caused by hydroxyl radical.

In the past decade, increasing numbers of chemical and biological studies on cdPu lesions have been performed. A recent review summarized the latest results that demonstrate the implications of cdPu in several areas, including DNA repair, biological effects, structural information, and the association with human diseases [13].

The repair of cdPu lesions, in both diastereomeric forms, has been thoroughly studied and attributed to nucleotide excision repair (NER) with different efficiency [13,14,15], i.e., the 5’*R* isomer being two times more efficient repair by NER than the 5’*S* isomer [15]. Molecular modeling and dynamics simulation elucidated that the different efficiency of NER is associated with the greater DNA backbone distortion caused by the 5’*R*isomer compared to the 5’*S* diastereomer [15,16] of the lesion. It has been found that these lesions are removed with a low efficiency by NER compared to other bulky DNA adducts [14], thereby leading to the accumulation of these oxidative lesions in the genome [1,14]. Consequently, this can result in the stalling of DNA polymerases at a DNA replication fork [17,18,19], further leading to an efficient bypass of a 5’*R*-cdA, but inefficient bypass of a 5’*S*-cdA by DNA polymerase β (pol β) during base excision repair (BER) [20,21]. This indicates that the DNA backbone distortions induced by the two diastereomeric cdPu lesions play a crucial role in modulating DNA repair efficiency.

In addition, it has been shown that 5’*S*-cdG also strongly inhibits transcription in vitro as well as in mammalian cells, thereby inducing transcriptional mutagenesis both in vitro and in vivo [4].

Moreover, the crystal structures of human polymerase η (Pol η) in bypassing 5’*S*-cdA was reported in low resolution. The study, indicates that DNA Pol η is the most efficient polymerase in inserting a nucleotide opposite a cdA, but fails to extend the modified nucleoside [22]. Another study has also shown elevated steady-state levels of cdPu lesions in mice lacking endonuclease VIII-like 1 (NEIL1), a DNA glycosylase that removes an oxidized DNA base lesion and initiates BER; this enzyme is unable to directly repair these lesions while it can cooperate with the NER pathway to repair the lesion [23]. Furthermore, it was reported that in *C. elegans*, an endonuclease III homolog (NTH-1) can bind to cdPu lesions, indicating that BER enzymes can bind to the lesions, but fail to remove them [13,24].

These findings have also shed light on the peculiar recognition of the 5’*S* in comparison with the 5’*R* lesion regarding DNA repair efficiency by NER or pol β efficiency and induction of transcriptional mutagenesis.

Moreover, it has been shown that there are a wide range of effects of cdPu lesions on DNA helicases that are key enzymes in processes that are either directly affected by DNA damage or are themselves implicated in the DNA damage response [25].

These results prompted us to investigate on the possibility that other DNA repair proteins may play a cooperative or facilitator role in the selective diastereomeric recognition. We focused on human poly(ADP-ribose) polymerase 1 (PARP1), in the recognition of 5’,8-cyclo-2’-deoxyadenosine (cdA), in both 5’*R* and 5’*S* diastereomeric forms, which significantly distorts the DNA backbone.

Indeed, PARP1, belonging to the 17-membered superfamily of PARPs, is an enzyme that is activated by the DNA damage response cascade and a regulator of DNA repair, including BER, homologous recombination, non-homologous end joining pathways as well as NER [26,27,28]. PARP1 can bind to single or double strand break intermediates [29,30]. It also plays an important role in gene transcription and chromatin remodelling in responding to distorted undamaged and damaged DNA structures, protecting the integrity of the genome and facilitating DNA repair. It has been indicated that PARP1 functions in the initial steps of damage recognition in global genome nucleotide excision repair (GG-NER), which is a dominant subpathway of NER.

Especially, PARP1 binds to the DNA damage-binding protein 2 (DDB2) and its interaction with DDB2 at chromatin impairment by UV radiation stimulates its catalytic efficiency [31,32]. It is shown that PARP1 can recognize an abasic site [33] and DNA lesions can distort the DNA backbone, such as a thymine dimer induced by UV irradiation [34]. However, it is unknown if the protein can recognize the four diastereomeric cdPu lesions that also distort the DNA backbone. Recent studies have shown that SIRT1 can be inhibited by the activation of PARP1, and this is strongly associated to NAD+ metabolism with the DNA damage responses through PARP1 [35,36]. This indicates that mitochondrial and mitophagic dysfunction through PARP-1 hyperactivation and NAD^+^/SIRT1 reduction may be implicated in XP-A patients’ neurodegeneration, revealing a progressive aging phenotype [37]. Furthermore, it has been indicated that CSB is a substrate for poly(ADP-ribosyl) ation by PARP-1 upon oxidative stress, implicating a functional interaction between PARP-1 and CSB in the response to oxidative damage [38]. It has been further demonstrated that stalled transcription at DNA secondary structures located in ribosomal DNA are associated with PARP1 activation in Cockayne syndrome and loss of CSA or CSB assembles on mitochondrial dysfunction [39]. In this study, we provide the first evidence that PARP1 binds to the 5’*R* and 5’*S* isomers of cdA lesions. We examined PARP1 binding to double stranded (ds) oligonucleotide (23 bases) DNA substrates containing 5’*R*- and 5’*S*-cdA diastereomers located at the sixth nucleotide from the 3′-end (Figure 1b). The affinity of binding between PARP1 and the substrates with the lesions were examined by distinct approaches, such as circular dichroism (CD), fluorescence spectroscopy, immunoblotting analysis, and gel mobility shift assay. We anticipate that PARP1 can interact both with the undamaged and the damaged substrates with incorporated cdA lesions in *R* and *S* diastereomeric form, exhibiting different affinities in binding to cdA lesions and undamaged DNA. The results provide new insights into the recognition and discrimination features of cdPu diastereomeric lesions by DNA repair machinery.

## 2. Materials and Methods

The radionuclides [γ-^32^P] ATP (6000 mCi/mmol) and Cordycepin 5’-triphosphate 3′-[γ-^32^P] (5000 mCi/mmol) were purchased from PerkinElmer Inc. (Boston, MA, USA). Human PARP1 (AG-40T-0011) and mouse PARP2 (AG-40T-0012) were purchased from Adipo Gen (Liestal, Switzerland). Both enzymes were purified by affinity chromatography (purity: ≥99% and ≥98%, respectively) and provided with a specific activity ≥600 U/mg protein.

All other chemical reagents and solvents were purchased from Link Technologies (Bellshill, Scotland, UK), Sigma Aldrich (Milan, Italy), Fluka (Seelze, Germany), and Carlo Erba (Cornaredo; Milan, Italy) and used as received. Deionized distilled water (Milli-Q) was used for HPLC and RP chromatography.

### 2.1. General Methods for Synthesis and Purification of Oligonucleotides (ODNs)

Oligonucleotides (ODNs) were prepared by automated synthesis using the (4,4’-dimethoxytrityl) protecting group (DMT-) and β-(cyanoethyl) phosphoramidite method, on CPG supports (500 Å), with an Expedite 8900 DNA synthesizer (Applied Biosystems, Monza MB, Italy) at the 1 µmol scale. Following the synthesis, the ODNs protected with DMTr-were cleaved from the solid support and deprotected by the method of two syringes using AMA reagent [NH_4_OH (30%)/CH_3_NH_2_ (40%) 1:1] for 10 min at room temperature. The AMA solution containing the cleaved ODN was placed in a sealed vial and heated for 15 min at 55 °C. The solvent was then removed in a Speedvac (Thermo Fisher Scientific, Monza (MB), Italy).

The crude 5’-DMT-on oligomers were purified and detritylated on-column by RP-HPLC (Grace Vydac C18 column 5 μm, 50 × 22 mm, Thermo Fisher Scientific, Rodano (MI), Italy).

The ODNs were further purified by SAX HPLC (preparative DNA Pac PA-100 column, 13 µm, 22 × 250 mm, Thermo Fisher Scientific, Rodano (MI), Italy).). The oligonucleotides were eluted with TRIS HCl 25 mM, pH = 8 (buffer A) and TRIS HCl 25 mM, NaClO_4_ 0.5 M, pH = 8.0 (buffer B) at a flow rate of 9 mL/min. The chromatographic method started with a gradient program from 2 to 30% B for 30 min, followed by an isocratic program with 30% B for 10 min, and a gradient step from 30 to 45% B for 5 min. The system was washed for extra 5 min with isocratic solution B 45% and an additional 10 min were given for re-equilibration after each analysis.

Elution of the ODNs was monitored at 254 nm. The purified ODN fractions were concentrated, desalted on Waters SepPak^TM^-C_18_-cartridges (Sesto San Giovanni (MI), and lyophilized.

The final yield of ODNs was estimated by UV absorption in aqueous solution at 254 nm by a Cary 100 UV/Vis Spectrometer (Agilent, Cernuscosul Naviglio (MI), Italy). The purified ODNs were then characterized by MALDI-TOF mass spectrometry (Voyager DE Pro, Applied Biosystems, Foster City, CA) and analytical SAX HPLC chromatography (DNA Pac PA100, 13 µm, 4 × 250 mm, Thermo Fisher Scientific, Rodano (MI), Italy).

### 2.2. Preparation of Double Stranded Oligonucleotide Substrates

The oligonucleotide strands 5’-d(GCA GAC ATA TCC TAG AGA CAT AT)-3′ (ss-N), or 5’-d(GCA GAC ATA TCC TAG AGX CAT AT)-3′ where X is 5’*S*-cdA for ss-5’*S* and 5’*R*-cdA for ss-5’*R*, were annealed to the complementary strands in equimolar concentrations in buffer solution containing 10 mM sodium phosphate, 100 mM NaCl, 0.1 mM EDTA, pH 7.2. The substrates were constructed by heating the two strands of the substrates at 90 °C for 10 min and subsequently allowing the temperature to slowly drop down to the room temperature (25 °C).

**Figure 2:**
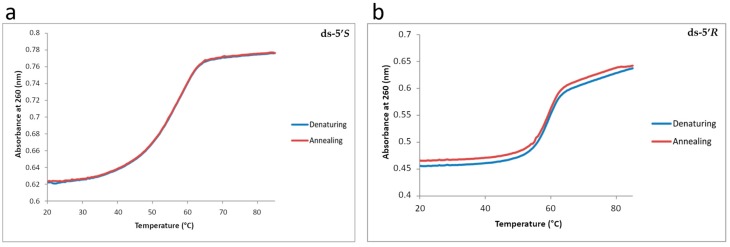
Ultraviolet (UV)melting curves of 23-mer duplexes containing (**a**) 5’*S*-cdA lesion and (**b**) 5’*R*-cdA lesion. UV melting curve of unmodified double stranded is shown in Figure S3.

Melting temperatures (Tm) of the substrates were measured with a Cary 100 UV/Vis spectrometer (Agilent, Cernuscosul Naviglio (MI), Italy) using a 1 mL quartz cuvette with a 1 cm path lenght. This allowed monitoring of the absorbance of the solutions at 260 nm as a function of the temperature. The temperature cycles were recorded from 20 to 80 °C per strand with a temperature controller at a heating rate of 0.3 °C/min (Table 1, Figure 2).

### 2.3. Circular Dichroism

CD spectra were recorded on a Jasco J-710 spectropolarimeter (Jasco-Europe, Cremella (LC), Italy) using a quartz cuvette (0.1 cm optical path length) at a scanning speed of 50 nm/min with 1 s response time. Measurements at the range of 200–360 nm were the average of four accumulations at 295 K and smoothed with Origin, Version 8.00 program (Origin Lab distributor Adalta, Arezzo, Italy). CD profiles of the binding of PARP1 and PARP2 proteins (15 μg·mL^−1^) to the increasing concentrations of double stranded oligonucleotide substrates with a dA or diastereomeric cdA lesions (50 nM, 100 nM, and 200 nM) were obtained in phosphate buffered saline (PBS buffer, 10 mM NaH_2_PO_4_, 100 mM NaCl, 0.1 mM EDTA, pH 7.2). The reported spectra are differential spectra, i.e., they were obtained by subtracting the spectrum of each oligo substrate from that obtained from the corresponding PARP-substrate complex. The secondary structures of PARP proteins were determined by the far-UV CD spectra using the CONTIN software package [40]. Each CD analysis was repeated three times. Ellipticity values were converted into molar ellipticity, Θ (deg·cm^2^·nmol^−1^), based on the molecular weight of PARP1 proteins.

### 2.4. Fluorescence Spectroscopy

Fluorescence spectra were recorded with an Edinburgh FLS 920 Fluorimeter (Hamamatsu Photonics, Arese (MI), Italy) continuous 450 W Xe lamp for excitation, equipped with a Peltier-cooled Hamamatsu R928(Hamamatsu Photonics, Arese (MI), Italy) photo multiplier tube for detection in right angle mode. Steady state fluorescence spectra of air-equilibrated solutions in the presence of PARP protein alone or the presence of PARP proteins and the substrates in PBS buffer under the same experimental conditions as those for measuring CD spectrum. The fluorescence was measured with selective excitation at 295 nm (excitation wavelength of tryptophan), in a small square cell (5 × 5 mm) [40]. All experiments were conducted in triplicate.

### 2.5. Immunoblotting Analyses of PARP Protein-DNA Complex

Polyacrylamide gel (8%) electrophoresis and immunoblotting were performed to detect the formation of PARP protein-DNA complex according to the method from Faraone-Mennella et al. [40]. The binding mixtures (9 μL) of PARP1 (0.15 μg) in the presence and absence of oligonucleotides were assembled in PBS buffer and diluted to 12 μL with sample buffer (60 mM Tris-HCl, pH 6.8, 1% SDS, 140 mM 2-mercaptoethanol, 20% glycerol, 0.15% bromophenol blue). PARP1-oligonucleotide complexes were shown to be stable under electrophoresis conditions, even in the presence of detergent and reducing agent, as described in [34,41,42]. Sample boiling was avoided.

Before loading, samples were incubated at room temperature in sample buffer, containing bromophenol blue, for 10 min. Gel electrophoresis was performed at 130 volt 25 mA for 40 min. PARP proteins were then electro transferred onto a PVDF membrane (0.2 μm Bio Rad) at 200 mA for 2 h at 4 °C in the electrophoresis buffer with 0.025% SDS. A polyclonal anti-PARP1 primary antibody in rabbit (H-250, Santa Cruz, CA, USA, 1:1000, *v*/*v*) was used to detect PARP1 proteins. The epitope corresponded to amino acids 764–1014 at the C-terminus of human PARP-1 (catalytic site). The primary antibodies, which bound to PARP1 proteins, were detected with a horseradish peroxidase (HRP)-conjugated goat anti-rabbit secondary antibody (IgG) from Bio-Rad Laboratory (Milan, Italy), which were subsequently incubated with the substrates producing HRP chemiluminescence (Super Signal West Dura Extended Substrate, 34075, PIERCE) that was measured by Chemidoc XRS (Bio Rad, Milan, Italy).

### 2.6. Gel Mobility Shift Assay

A double stranded DNA substrate with a dA or diastereomeric cdA lesions opposite T in the template strand was employed to measure the binding of PARP1 to the DNA bases. The substrate was created by annealing a DNA strand with a dA or cdA to a complementary strand at a molar ratio of 1:2. Substrates were radiolabeled at the 5’-end of the strand containing a dA or cdA. Substrates (10 nM) were incubated with various concentrations of PARP1 (30, 40, and 50 nM) in binding buffer containing 50 mM Tris-HCl, pH 7.5, 50 mM KCl, 0.1 mM EDTA, 0.1 mg/mL bovine serum albumin, 0.1% Nonidet P-40, and 5% glycerolon ice for 8 min. PARP1-DNA complex was then separated from free DNA by agarose (1%) acrylamide (0.1%) gel electrophoresis and detected by a Pharos FX Plus Phosphor Imager from Bio-Rad (Hercules, CA, USA).

## 3. Results and Discussion

### 3.1. Synthesis and Characterization of 23-Mer Oligonucleotides Containing dA and cdA Lesions

The phosphoramidites of the two 5’*R* and 5’*S*-cdA nucleosides were prepared following the radical-based protocols developed previously [43]. The modified and unmodified23-mer oligo-2’-deoxyribonucleotide (ODN) sequence 5’-d(GCA GAC ATA TCC TAG AGX CAT AT) with X = 5’*R*- or 5’*S*-cdA or dA were synthesized by automated synthesis and purified following the procedures described previously [44]. More specifically, after standard deprotection with AMA reagent (NH4OH (30%)/CH3NH2 (40%)), the crude 50-DMTr-on ODNs were detritylated and purified by reversed phase HPLC. Further purification was carried out by strong anion-exchange (SAX). HPLC and the purity and homogeneity of the collected fractions was monitored by analytical strong anion-exchange (SAX) HPLC (Figure S1). The molecular weights of the ODNs were assessed by MALDI-TOF in the negative mode (Table 2, Figure S2).

### 3.2. Thermal Stability of Modified Oligonucleotide Duplexes by Diastereomeric cdA Lesions

The thermal stabilities of the 23-mer duplexes 5’-d(GCA GAC ATA TCC TAG AGX CAT AT) •3’-d(CGT CTG TAT AGG ATC TCT GTA TA) with X = 5’*R*- or 5’*S*-cdA or dA were obtained by analysis of the UV melting profiles. Typical UV melting profiles are shown in Figure 2 (panels a, b) and the melting points of the duplexes are summarized in Table 1. Both cdA lesions destabilize the 23-mer duplexes as shown by the differences in the melting points (Tm), ΔTm = Tm (modified)—Tm (unmodified). The ΔTm values are near −1 and −2 °C in the case of duplexes with either the 5’*R*-cdA or the 5’*S*-cdA lesions in the 23-mer duplex sequence context investigated (Table 1). Analogousde stabilizations due to cdPu lesions have been observed previously in different sequence contexts and oligomer lengths [14,45,46].

### 3.3. PARP1 Recognizes an Undamaged dA and cdA Diastereomeric Lesions in ds-Oligonucleotides

**Figure 3:**
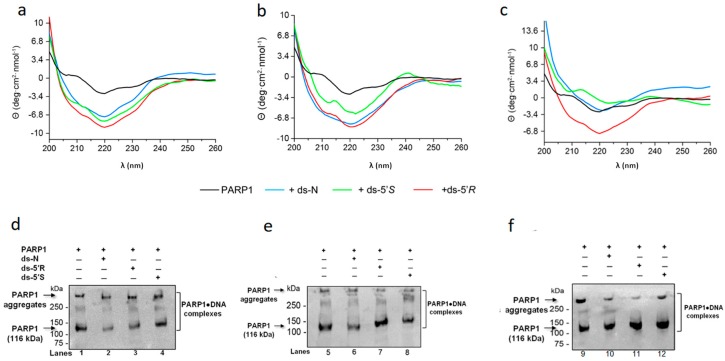
Circular dichroism and immunoblotting analysis for determining PARP1 binding to ds-oligonucleotides with cdA lesions. (**a**–**c**) CD profiles of PARP1 without or with the substrates. Human PARP1 protein only (15 μg·mL^−1^, black line), PARP1 along with 50 nM (**a**), 100 nM (**b**), or 200 nM (**c**) ds-N (blue line), or ds-5’*S* (green line) or ds-5’*R* (red line), respectively. (**d**–**f**) Immunoblotting results of PARP1 in the presence of 50 nM (**d**), 100 nM (**e**), and 200 nM (**f**) oligonucleotide substrate. Lanes 1, 5, and 9 correspond to PARP1 only. Lanes 2, 6, and 10 correspond to PARP1 with 50 nM, 100 nM, and 200 nM ds-N, respectively. Lanes 3, 7, and 11 correspond to PARP1 with 50 nM, 100 nM, and 200 nM substrates containing a ds-5’*R*-cdA, respectively. Lanes 4, 8, and 12 correspond to PARP1 with 50 nM, 100 nM, and 200 nM substrates containing a ds-5’*S*-cdA, respectively. The results in the panels d-f are from different immune blottings; the blots are shown in Figure S5.

Since PARP1 can interact with a DNA substrate without a lesion [47,48] and the ends of ds DNAs, this makes it challenging to differentiate its binding to the cdA lesions from the binding to the ends. To solve this problem, we employed circular dichroism (CD) to determine the binding of PARP1 to the cdA lesions. We reason that the binding of PARP1 to cdA lesions may allow PARP1 to adopt different secondary structures from its binding to undamaged DNA and ends of the ds DNAs. Using this approach, we initially determined the secondary structure of PARP1, in the absence and presence of ds-oligonucleotides, by following the absorbance of the peptide bond in the “far-UV” spectral region of 190–250 nm (Figure 3). The spectrum of each oligonucleotide was subtracted from that of the corresponding PARP1/oligo complex to illustrate the effects from the protein alone. Changes in CD signals resulting from the protein-DNA complex were used to determine the affinity of the PARP1-oligonucleotide interaction as well as to provide information about the nature and conformational changes of PARP1 protein. In addition to the measurement of the differences in the trends of spectra, two parameters of protein CD spectra, the variability of negative ellipticity minimum and the possible wavelength shift of the minimum, were also measured [49].

The PARP1 binding to the ds oligonucleotide substrate without or with a 5’*S*- or 5’*R*-cdA (ds-N, ds-5’*S*, ds-5’*R*) was determined by incubating the substrates at 50 nM, 100 nM, and 200 nM with PARP1 (0.13 nM).The changes in CD signals upon the presence of both PARP1 protein and the oligonucleotide substrates indicate the interaction between PARP1 and damaged and undamaged DNA sequences. We observed that this interaction was different in function of the substrate used, as shown by the profile of the CD spectra (Figure 3a–c) and the trend of the ellipticity minimum resulting from the different substrates (Table 3). The results indicate that PARP1 bound to cdA lesions by adopting a different secondary structure from its binding to dsDNA.

The CD spectra showed that at almost all concentrations of the substrates, except for the 200 nM ds-5’*S*substrate, the binding of PARP1 to the substrates led to a beta conformation of protein, indicating that the substrates were able to stabilize the structure of PARP1 protein (Figure 3a–c, Table 3). At 200 nM, only ds-5’*R* maintained unchanged the beta conformation of PARP1, which was highly stabilized. Thus, PARP1 interacted differently with the two diastereoisomeric lesions.

For all substrates at 50 nM, the negative ellipticity minimum was very close to each other, but it exhibited a trend towards the positive values with increasing concentrations of all substrates (Table 3).

The ds-5’*R*-cdAcontaining substrate reduced the variation of the negative ellipticity minimum much less than the other substrates, resulting in the best arrangement of the PARP1 spectrum (Figure 3a–c, Table 3). The results indicate that PARP1 can also bind to both 5’*R* and 5’*S* diastereomeric cdA lesions.

To further evaluate PARP1 interaction with the substrates, an aliquot of the mixtures used for CD spectra was loaded on a SDS-polyacrylamide gel. PARP1 protein in the protein-oligonucleotide complex was immunoblotted and analysed by an anti-PARP1 antibody (Figure 3d–f). The results showed that PARP1 protein alone exhibited a different mobility from PARP1-oligonucleotide complexes.

Since control ds-N and ds-5’*S* or -5’*R* oligonucleotides differ only for the cdPu lesions, we reason that any increase in PARP1 binding to the substrate containing a cdA lesion, compared to control dsDNA, may be due to the binding of PARP1 to the lesions. In fact, the mobility of the complexes’ protein-oligonucleotide with a cdA lesion in the gel was retarded compared to N, and the formation of complexes at a higher molecular weight was detected (Figure 3d, lanes 2–4; Figure 3e lanes 6–8 and Figure 3f lanes 10–12). The broadening bands in the gels suggested the formation of various species of complexes of PARP1-oligonucleotide with various intermediate conformations of PARP1 proteins.

Thus, the complexes of PARP1-cdA substrates exhibited different gel shifts from the complexes of PARP1-dsDNA.

The results of immunoblotting analysis confirmed PARP1 binding to the cdA lesions.

**Figure 4:**
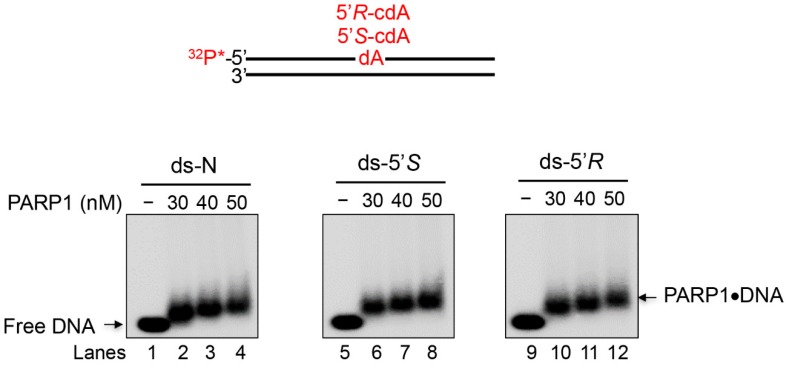
Gel mobility shift assay for determining PARP1 binding to ds-oligonucleotides with cdA lesions. Lanes 1, 5, and 9 correspond to the substrate only. Lanes 2, 6, and 10 correspond to the binding mixture with 30 nM PARP1. Lanes 3, 7, and 11 correspond to the binding mixture with 40 nM PARP1. Lanes 4, 8, and 12 correspond to the binding mixture with 50 nM PARP1. The DNA substrates are schematically illustrated above the gel. Grouping of gels are cropped from different parts of the same gel. The original gel is represented in Figure S6. The gel mobility shift assay procedure is described in the Materials and Methods in the main text. * represents a phosphate group.

In order to further confirm PARP binding to cdA lesions, we examined the PARP1 binding to ds-N, ds-5’*S*, ds-5’*R*, by ^32^P-labelled DNA gel mobility shift assay at various concentrations of PARP1 (30, 40, and 50 nM). This assay was useful to understand the behaviour of the DNA counterpart, confirming the recognition of all three substrates by PARP1 and the formation of PARP1-oligonucleotide complexes (Figure 4 and Figure S6 for full-length gel images). However, it is worth noting that this assay has only a qualitative meaning, whereas it cannot appreciate significant differences in the binding of the undamaged and damaged substrates.

### 3.4. PARP1 Binding to Undamaged dA and cdA Lesions with Different Affinities

**Figure 5:**
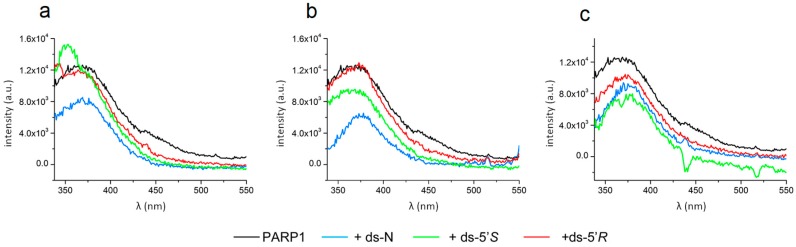
Intrinsic fluorescence of PARP1 in the absence and presence of DNA substrates. (**a**–**c**) The fluorescence profiles of PARP1 protein alone (15 μg·mL^−1^, black line) or that of PARP1 along with 50 nM (**a**), 100 nM (**b**), and 200 nM (**c**) of ds-N (blue line) or ds-5’*S* (green line) or ds-5’*R* (red line).

To further confirm the PARP1 binding to the substrates with or without cdA lesion, fluorescence spectroscopy was employed to determine the formation of the PARP1-oligonucleotide substrate complex. The binding of the protein to the substrates was determined by measuring the quenching of intrinsic PARP1 fluorescence. This is because the intrinsic fluorescence of tryptophan residues in PARP1 proteins can be quenched by DNA bases when the protein forms a complex with a DNA substrate, that can also modulate the local environment of the fluorophore, such as the changes of hydrophobicity of the microenvironment. A hydrophobic environment increases the fluorescence intensity from a tryptophan, an aromatic and hydrophobic amino acid, whereas the fluorescence decreases if tryptophan moieties are exposed to a hydrophilic environment. Thus, this method can also be used to determine the conformational change of PARP1 protein within its complex with DNA and the binding affinity of the protein to DNA. In Figure 5, the fluorescence intensity of PARP1 in the presence of ds-5’*R* substrate at 50 and 100 nM (panels a and b) overlapped that of PARP1 alone, although the maximum value tends to shift towards a longer wavelength (lower frequency). This indicated that the binding of PARP1 to the substrates did not significantly alter the hydrophobic environment of the fluorophore Trp, although inducing a change by increasing the wavelength of the maximal intensity (blue shift). The decrease of fluorescence intensity appeared at the 200 nM ds-5’*R* substrate (Figure 5c). Thus, on oligo binding, perturbation of the Trp environment was limited, but was enough to allow a PARP1 conformational change. In fact, these results are in line with those of Figure 3a–c, where the arrangement of the PARP1 secondary structure to get a beta conformation took already place at the 50 nM and 100 nM ds-5’*R* substrate, and was stable up to the 200 nMds-5’*R* substrate.

For ds-5’*S* at a low concentration, 50 nM (Figure 5a), PARP1 exhibited a similar binding to its binding to the ds-5’*R* substrate. At a high concentration of *N*, *S* substrates (Figure 5c), the binding of PARP1 led to exposure of the fluorophores to a more hydrophilic environment, with a decreased fluorescence intensity, and a high perturbation of the Trp surroundings. This is consistent with the results in Figure 3b showing that ds-5’*S* and N substrates were less efficient than ds-5’*R* to stabilize PARP 1 beta-conformation.

The intrinsic fluorescence intensity altered by the formation of PARP1-oligonucleotide complex allowed us to calculate the affinity constants (*K_a_*) of PARP1 binding to different substrates with a cdA lesion at a saturating concentration of the substrates, which are shown in Table 4.

The results showed that PARP1 bound to all, damaged and undamaged, substrates with the same affinity at a saturating concentration (10^7^ order of magnitude), except for ds-5’S, which is two orders of magnitude higher than the others (10^9^ order of magnitude).

Considering that both damaged substrates reach saturation at 200 nM and induce a qualitatively comparable conformational change of PARP1, it is conceivable that the binding affinity of ds-5’*S* might be due to a different way of interaction with the protein.

To further explore the role of the zinc finger domain of PARP1 in mediating its binding to the undamaged and damaged substrates with cdA lesions, we examined the binding of mouse PARP2 to the substrates. Since PARP2 does not have a zinc finger, the difference of the substrate binding of PARP1 and PARP2 would indicate if the zinc finger of PARP1 is involved in the substrate binding and recognition of the cdA lesions. It should be noted that both PARP1 and PARP2 have two C-terminal domains—Trp-Gly-Arg (WGR) and catalytic (CAT) regions. The N-terminal region (NTR) of PARP1 contains over 500 amino acid residues with four regulatory domains, including three zinc-fingers that are essential for DNA-dependent PARP1 activity, whereas PARP2 has a small NTR (70 residues) and lacks the zinc fingers. However, the region is enriched with basic amino acid residues that may serve as a potential DNA binding domain [50,51]. A previous study has shown that the basic amino acid residues within the NTR of this protein exhibited a potential DNA-binding ability [48].

However, recent structural and biochemical studies have shown that the WGR domain of PARP1 plays a central role in coordinating with allosteric regulation of the CAT domain as well as with Zn1 and Zn3 domains [52,53]. Since a recent study from the Pascal group suggests that PARP2 can be activated in response to specific DNA repair intermediates, such as the nicked DNA [29], we then explored the binding of mouse PARP2 to the undamaged DNA substrate and the substrates containing a cdA lesion (Figure S4). The results of fluorescence and CD spectroscopy showed that the binding of PARP2 to the 5’*R*-cdA diastereomer resulted in an increase of fluorescence intensity and the blue shift of the negative ellipticity minimum wave length, indicating that the 5’*R*-cdA isomer stabilizes the fluorophore hydrophobic environment. This further suggests that the lesion stabilized the conformation of PARP2, an unexpected indication as it is well considered that PARP2 NTR is disordered. The last evidence strongly suggests that PARP2 interacts with the 5’*R*-cdA in its native conformation. In contrast, opposite results were obtained from the binding of PARP2 to a 5’*S*-cdA lesion, revealing that PARP2 bound to 5’*S*-cdA in a different manner from a 5’*R*diastereomer.

The obtained diverse PARP2 interaction with diastereomeric cdA lesions suggest that the zinc finger domain of PARP1 is not required for sustaining its complex with a substrate containing a cdPu lesion. This has further paved the way for further studies on the function of mutant forms of PARP1 or different PARP domains in mediating the binding of PARP proteins with DNA substrates.

## 4. Conclusions

In this study, we characterized the binding affinity and recognition patterns by the PARP1 protein of 5’*S*-cdA and 5’*R*-cdA lesions, unique DNA adducts associated with the NER pathway, in double stranded oligonucleotide substrates. It must be underlined that the presented results help to understand the structural interactions of PARPs with modified oligonucleotides, which is the novelty of this work, but further studies are required for proposing any functional role of these enzymes. Our finding for the first time revealed the ability of PARP1 to recognize in vitro the peculiar DNA backbone distortion and differentiate the diastereomeric forms of a cdA lesion, as detected by different approaches at distinct oligonucleotide sequences, with a higher affinity constant for the 5’*S* lesion in a mimetic model of dsDNA.

## Figures and Tables

**Table 1:** Melting points, Tm, of 23-mer DNA duplexes.

5′-d(GCA GAC ATA TCC TAG AGX CAT AT)-3′ 3′-d(CGT CTG TAT AGG ATC TCT GTA TA)-3′	Tm, °C
X = dA (unmodified)	60.0 ± 0.3
X = 5’*R*-cdA	59.0 ± 0.2
X = 5’*S*-cdA	58.0 ± 0.3

**Table 2:** Sequences and molecular masses of the synthesized ODNs.

Strands	Sequence (5’–3′) ^1^	Mass Calcd. (Da)	Mass Found ^2^ (Da)
ss-N	GCA GAC ATA TCC TAG AGA CAT AT	7040.7	7038.1
ss-5’*S*	GCA GAC ATA TCC TAG AGX CAT AT	7038.7	7037.2
ss-5’*R*	GCA GAC ATA TCC TAG AGX CAT AT	7038.7	7036.9
CS ^3^	ATA TGT CTC TAG GAT ATG TCT GC	7044.7	7044.7

^1^ X is 5’*S*-cdA for ss-5’S and 5’*R*-cdA for ss-5’*R*. ^2^ All the oligonucleotide masses were obtained by MALDI-TOF in negative mode. [M − nH]^−^. ^3^ CS = Complementary strand.

**Table 3:** Ellipticity values were converted into molar ellipticity Θ (deg·cm^2^·nmol^−1^) based on the molecular weight of PARP1 proteins, of human PARP1 alone and along with increasing concentrations (50 nM, 100 nM, 200 nM), of double stranded oligonucleotides (ds-N, ds-5’*S*-cdA, ds-5’*R*-cdA), respectively, at 220 nm. The results are illustrated as mean values of triplicate with errors ± 5%.

Compounds	Θ_220nm_ 50 nM	Θ_220nm_ 100 nM	Θ_220nm_ 200 nM
PARP1	−2.84	−2.84	−2.84
PARP1 + ds-N	−7.14	−7.79	−2.46
PARP1 + ds-5’*S*	−7.96	−5.82	−0.53
PARP1 + ds-5’*R*	−9.09	−8.26	−7.26

**Table 4:** Affinity constants (*K_a_*) of PARP1 binding to substrates.

Substrates	Saturation at (nM)	*K_a_* * (M^−1^)
ds-N	100	1.51 × 10^7^
ds-5’*S*	200	3.19 × 10^9^
ds-5’*R*	200	1.21 × 10^7^

* Details of constant calculations at a saturating concentration of substrates are illustrated in Supporting Information (pages S7 and S8).

## Supplementary Materials

The Supplementary Materials are available online at http://www.mdpi.com/2073-4409/8/2/116/s1.
