# Supplementary material for: Diastereomeric Recognition of 5’,8-cyclo-2’-Deoxyadenosine Lesions by Human Poly(ADP-ribose) Polymerase 1 in a Biomimetic Model
Source: Cells. 2019 Feb 2;8(2):116. doi: 10.3390/cells8020116 (PMC6406461; doi:10.3390/cells8020116)
Supplement: Supplementary file 1 [file cells-08-00116-s001.pdf]

cells

# Diastereomeric Recognition of 5′,8-cyclo-2′-Deoxyadenosine Lesions by Human Poly(ADP-ribose) Polymerase 1 in a Biomimetic Model

**Annalisa Masi[1*], Arianna Sabbia[2], Carla Ferreri[1*], Francesco Manoli[1], Yanhao Lai[3], Eduardo Laverde[4], Yuan Liu[3,4,5], Marios G. Krokidis[6], Chryssostomos Chatgilialoglu[1], Maria Rosaria Faraone Mennella[2*]**
**Annalisa Masi [1,*,†], Arianna Sabbia [2], Carla Ferreri [1,*,†], Francesco Manoli [1], Yanhao Lai [3], Eduardo Laverde [4], Yuan Liu [3,4,5], Marios G. Krokidis [6], Chryssostomos Chatgilialoglu [1] and Maria Rosaria Faraone Mennella [2,*,†]**

1 Istituto per la Sintesi Organica e la Fotoreattività, Consiglio Nazionale delle Ricerche, Bologna 40129, Italy; francesco.manoli@isof.cnr.it (F.M.); chrys@isof.cnr.it (C.C.)
2 Dipartimento di Biologia, Università di Napoli "Federico II", 80138 Napoli, Italy; ar.sabbia@studenti.unina.it
3 Department of Chemistry and Biochemistry, Florida International University, Miami, FL 33199, USA; yalai@fiu.edu (Y.L.); yualiu@fiu.edu (Y.L.)
4 Biochemistry Ph.D. Program, Florida International University, Miami, FL 33199, USA; elave006@fiu.edu
5 Biomolecular Sciences Institute, Florida International University, Miami, FL 33199, USA
6 Institute of Nanoscience and Nanotechnology, NCSR Demokritos Agia Paraskevi, 15310 Athens, Greece; m.krokidis@inn.demokritos.gr
* Correspondence: annalisa.masi@isof.cnr.it (A.M.); carla.ferreri@isof.cnr.it (C.F.); faraone@unina.it (M.R.F.M.); Tel.: +39 051 6398313 (A.M.); +39 051 6398289 (C.F.); +39 081 679136 (M.R.F.M.)
† These authors contributed equally to the work.


Received: 21 December 2018; Accepted: 1 February 2019; Published: 2 February 2019

**Table of contents:**

Starting materials S3

ODNs characterization: HPLC, MALDI (Figures S1 and S2) S3-S6

Melting point experiments (Figure S3) S6

Calculations of affinity ($K_a$) and dissociation ($K_d$) constants S7-S8

Circular dichroism and fluorescence analyses for PARP2 (Figure S4) S9

Full-length immunoblottings and gel mobility shift assay (Figures S5 and S6) S10-S11

**Starting materials for oligonucleotide synthesis:** Chemicals were purchased from Sigma Aldrich, Fluka and Link Technologies. HPLC-grade acetonitrile was obtained from Sigma Aldrich. Buffer for high performance liquid chromatography was prepared using water purified with a Mill-Q system. MALDI-TOF mass spectrometry and analytical SAX HPLC chromatography were used to characterize the purified ODNs.

**Oligonucleotides (ODNs) characterization:**

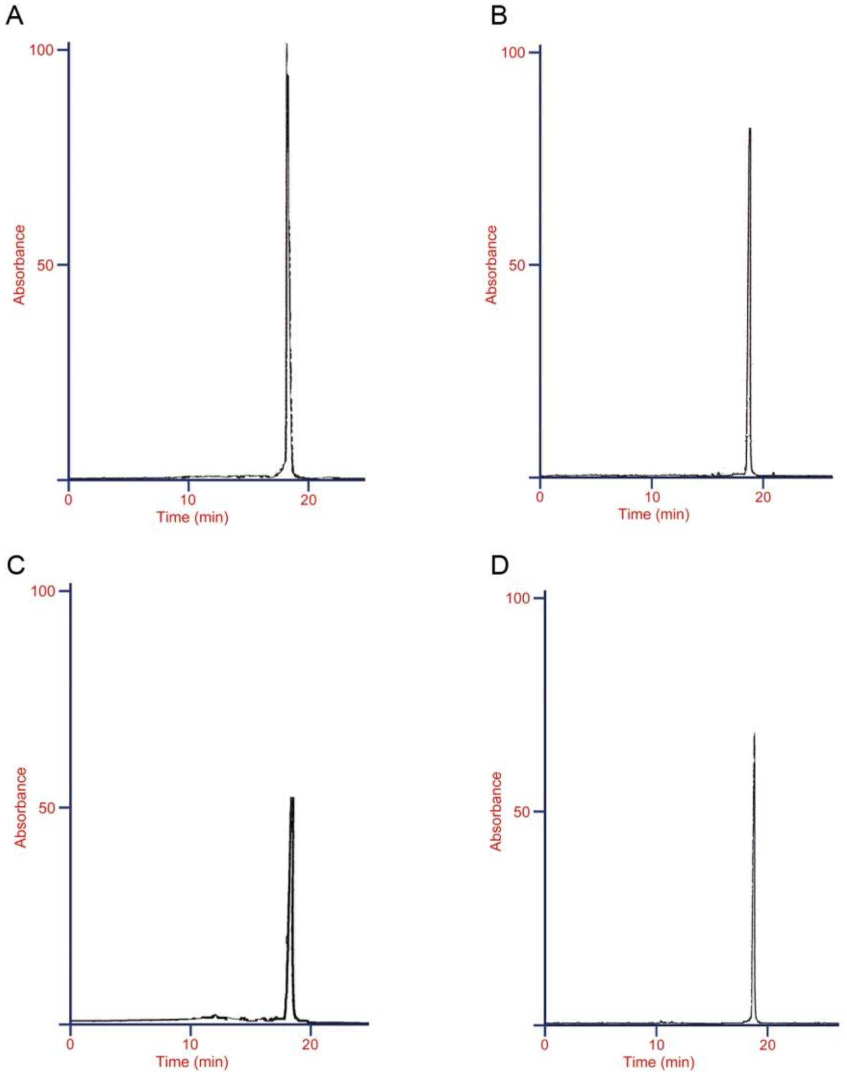


**Figure S1. Analytical SAX HPLC chromatograms of purified ODNs.** Column and conditions: SAX DNAPac PA-100 column, 5μm, 4x250 mm; mobile phase A: TRIS·HCl 25 mM, pH 8.0, mobile phase B: TRIS·HCl 25mM, $NaClO_4$ 0.5M, pH 8.0. Gradient: 2-30 % B in A in 30 min. Flow rate was 1 mL/min. (A) ss-N, (B) ss-5′*S*, (C) ss-5′*R*, (D) complementary strand.

**Molecular masses of the synthesized ODNs** All the oligonucleotide masses were obtained by MALDI-TOF in negative mode.

**ssN:** GCA GAC ATA TCC TAG AGX CAT AT, X=dA; Mass calcd Da 7040.7; Mass Found Da 7038.1.

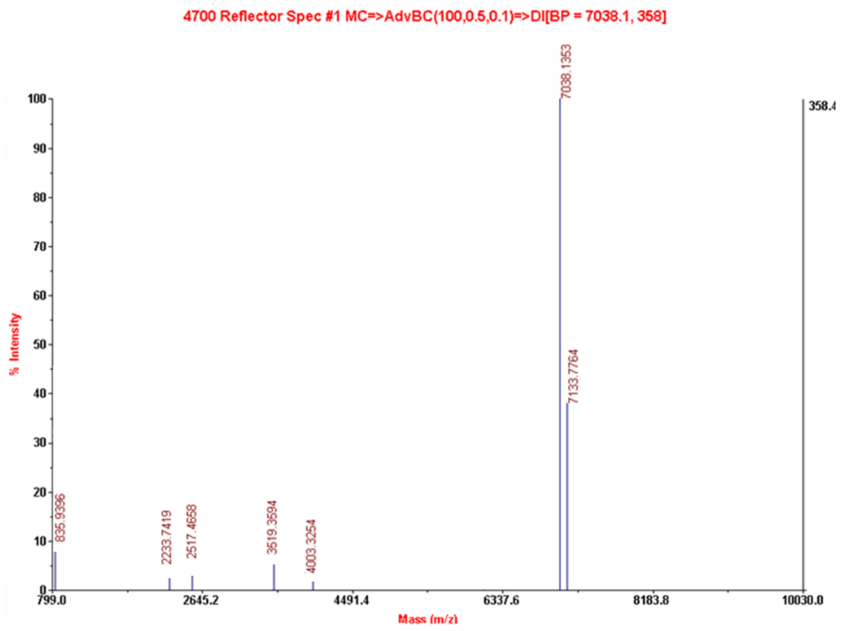


**ss-5′S:** GCA GAC ATA TCC TAG AGX CAT AT, X=5′*S*-cdA; Mass calcd Da 7038.7; Mass found Da 7037.2

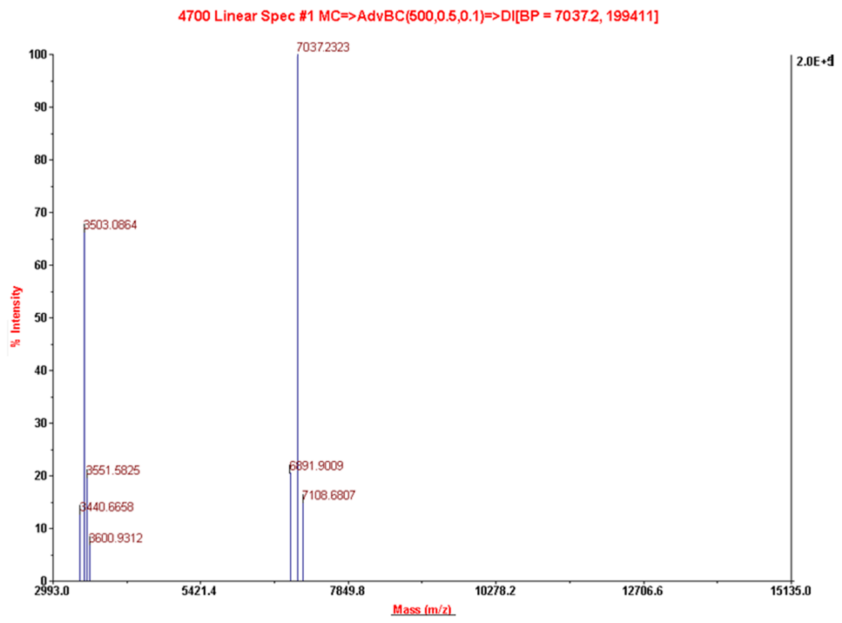

**ss-5′R:** GCA GAC ATA TCC TAG AGX CAT AT, X=5′*R*-cdA; Mass calcd Da 7038.7; Mass found Da 7036.9

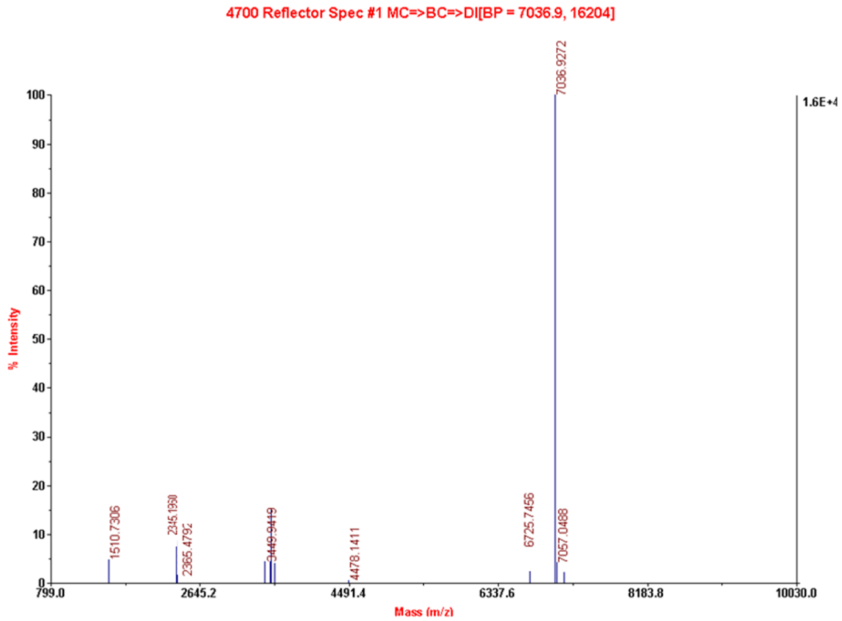


**CS=complementary strand** ATA TGT CTC TAG GAT ATG TCT GC; Mass calcd Da 7044.7; Mass found Da 7044.7

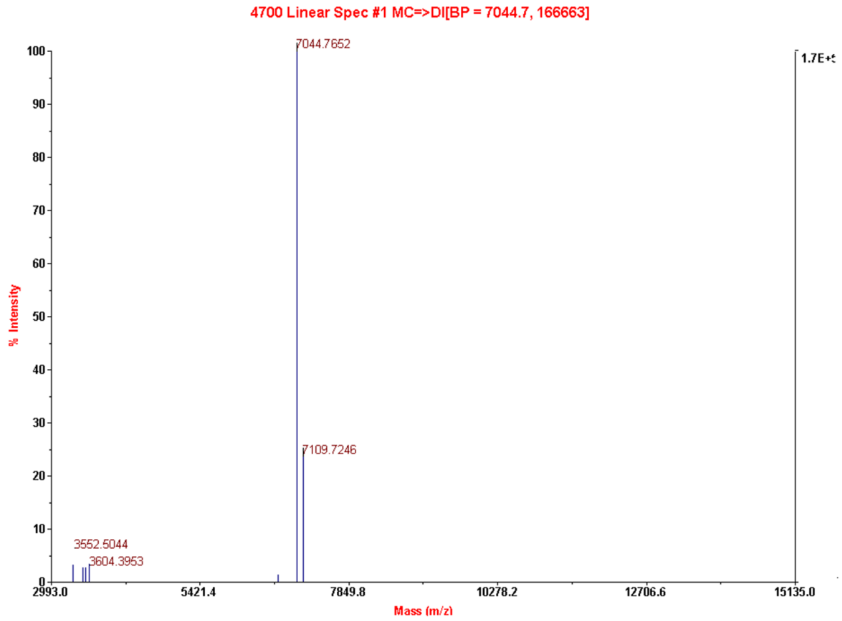


**Figure S2 MALDI-TOF analyses.** The analyses were performed using a Voyager DE Pro (Applied Biosystems, Foster City, CA) equipped with a pulsed N2 laser operating at 337 nm. Whole oligonucleotides negative ion spectra were acquired in linear mode over a m/z range from 3500 to 7000 using a 20000-V accelerating voltage, a 17000-V grid voltage, and a delay extraction time of 200 ns. The spectrum for each spot was obtained by

averaging the result of 100 laser shots. External mass calibration was performed using peptides standard mixture (mass range: 1000-6000). The analyses were performed by spotting on the target plate 1 μL of the sample mixed with an equal volume of the matrix solution, 50 mg/mL 3-hydroxypicolinic acid/ 50 mg/mL diammonium citrate (9/1) (v/v) in water.

**A: Melting Temperature of ds-N:**

5′-d(GCA GAC ATA TCC TAG AGA CAT AT)-3′

5′-d(ATA TGT CTC TAG GAT ATG TCT GC)-3′

**Tm** = 60°C

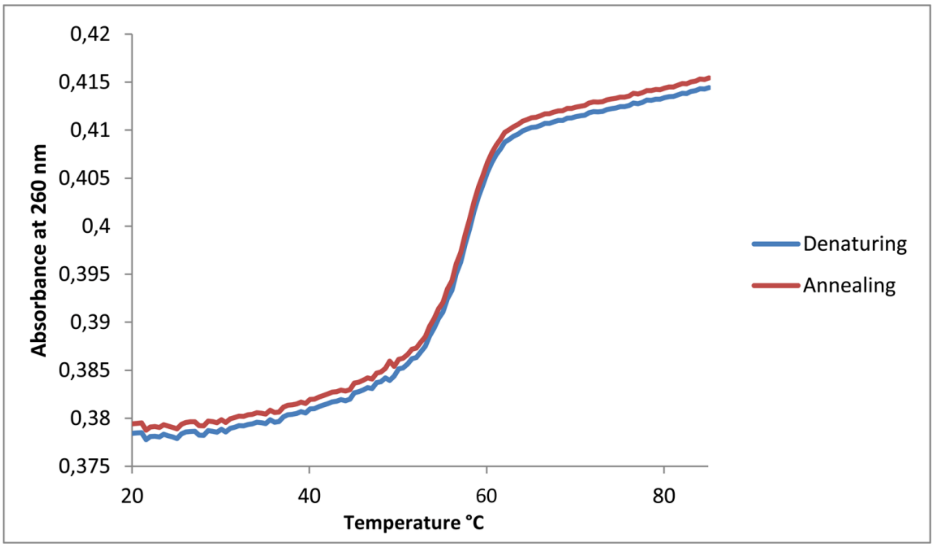


**Figure S3** UV melting curves of 23-mer duplexes. Melting temperatures (Tm) of the substrates were measured with a Cary 100 UV/Vis spectrometer using a 1 mL quartz cuvette with a 1 cm pathlenght. This allowed to monitor the absorbance of the solutions at 260 nm as a function of temperature. The temperature cycles were recorded from 20 to 80 °C per each strand with a temperature controller at a heating rate of 0.3 °C/min

**Calculations of affinity (*$K_a$*) and dissociation (*$K_d$*) constants of PARP1/oligonucleotide binding at saturating concentration of ligands.**

Preliminary calculations

The following parameters were used for calculation:

**Q =** fluorescence quenching , at 369nm (max intensity of free PARP1)

$$Q = \frac{Io - I}{Io}$$

**$I_0$** = max fluorescence intensity of free PARP1= 12630 (fluorescence intensity)

**I**= fluorescence intensity of free PARP1 in the presence of oligonucleotide at concentration of: 50, 100 or 200 nM

**[total PARP1]**=59,17·$10^{-9}$M (1µg/130 µl)

$$[bound\ PARP1] = [total\ PARP1] \times Q$$

$$[free\ PARP1] = [total\ PARP1] - [bound\ PARP1]$$

Constant calculations

The constant was calculated by McGhee & von Hippel equation at saturating concentration of ligands

Binding of PARP1 with ds-N was non cooperative (represented by a hyperbole, n=1).

Binding of PARP1 with ds-*S* and ds-*R* was cooperative (represented by sigmoids), with the number of binding sites (n =2) as calculated by Hill equation:

$$\theta = \frac{[ligand] \times n}{[[ligand] \times n + \mathrm{K}_{0.5}}$$

where: $K_{0.5}$=[ligand] at 50% of engaged sites.

$$\frac{\nu}{[free\ ligand]} = kd \times (1 - \mathrm{n}\nu)\left[\frac{1 - \mathrm{n}\nu}{1 - (\mathrm{n} - 1)\nu}\right]^{n-1}$$

Where:

$$\boldsymbol{\nu} = \frac{[bound\ ligand]}{[total\ protein]}$$

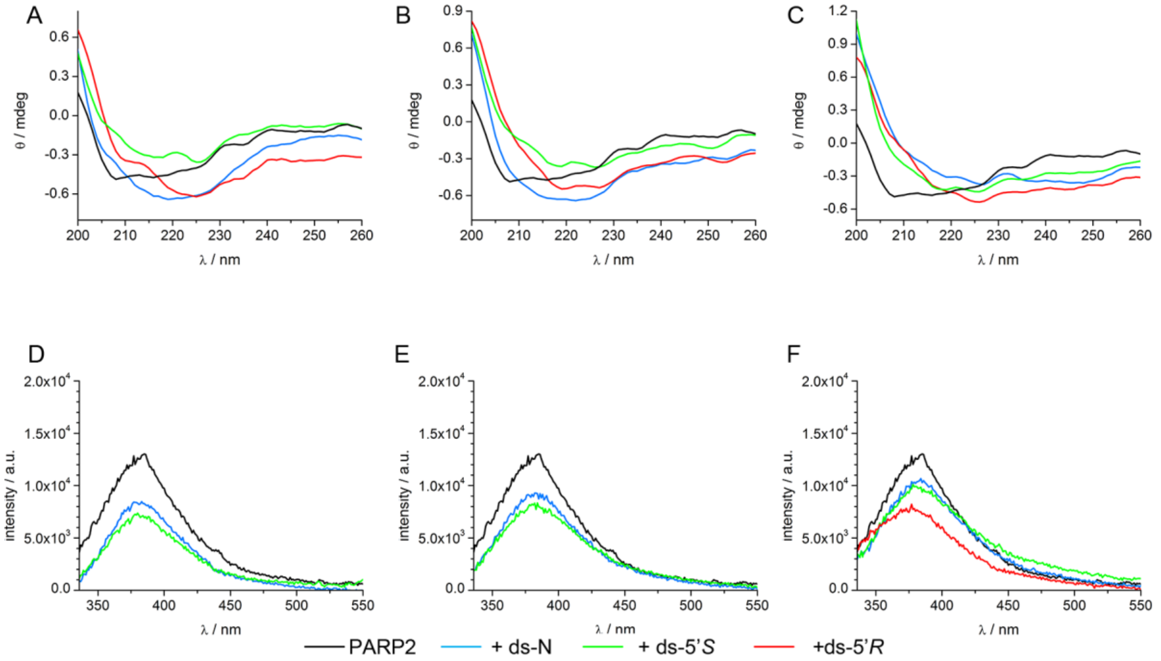


**Figure S4. Circular dichroism and intrinsic fluorescence of mouse PARP2 in the absence and presence of ds-oligonucleotide substrates.** (A-C) The CD profiles of mouse PARP2 alone (15 μg mL$^{-1}$,AG-40T-0012, AdipoGen, *black line*) and that of PARP2 along with 50 nM (A) or 100 nM (B) or 200 nM (C) ds-N (*blue line*), or ds-5′*S* (*green line*) or ds-5′*R* (*red line*), respectively. The profiles were measured in PBS buffer as described in Methods. (D-F) Experimental fluorescence profiles of Mouse PARP2 alone (15 μg·mL$^{-1}$, *black line*) and that from PARP2 along with 50 nM (D) or 100 nM (E) or 200 nM (F) of ds-N (*blue line*), or ds-5′*S* (*green line*) or ds-5′*R* (*red line*).

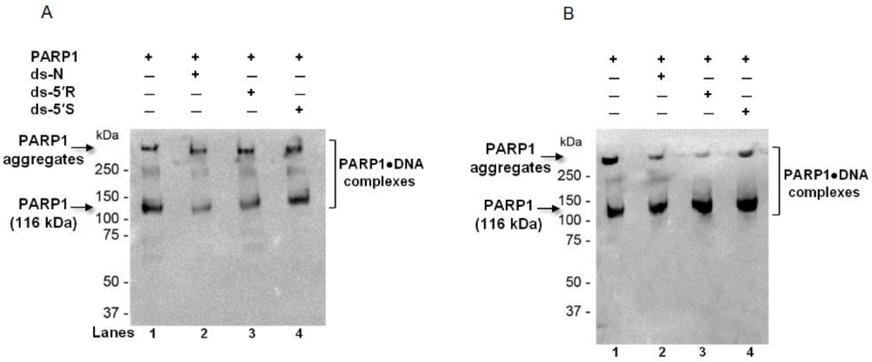


**Figure S5. Full-length of immunoblotting analyses of Figure 3.** (A, B) Full-length immunoblotting of PARP1-oligonucleotide complexes in the presence of 50 nM (A) and 200 nM (B) of double stranded oligonucleotide substrates. Each panel is a different blot. The full-length immunoblots evidence that only PARP is involved in the interaction with oligonucleotides. Immunoreactive bands start at native molecular weight of PARP and appear above, up to the top of the filter (aggregates).

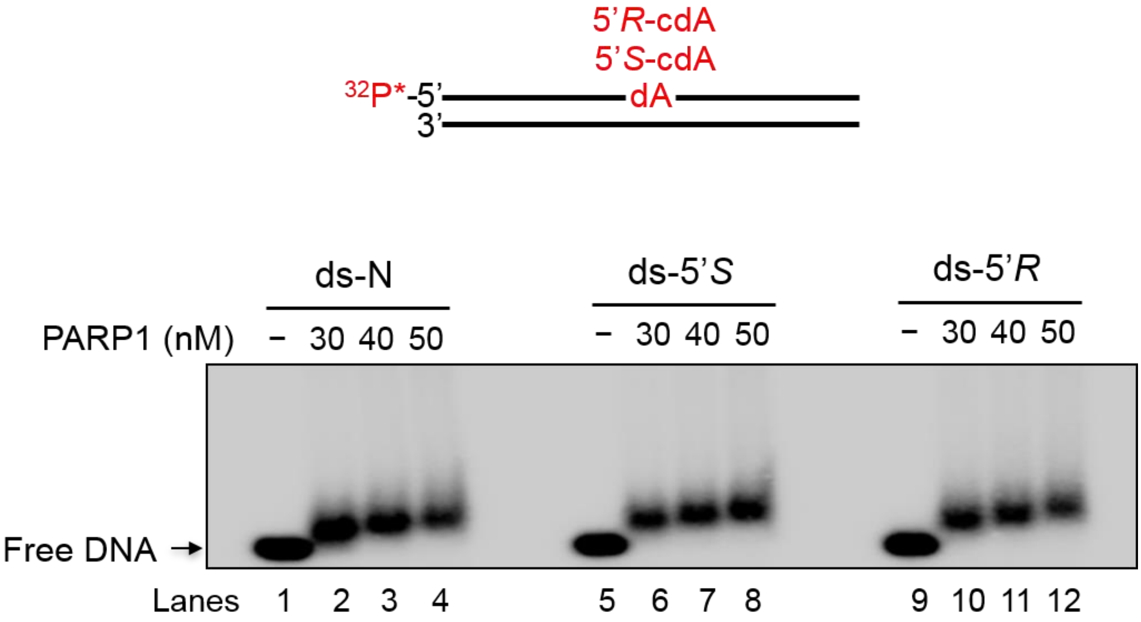


**Figure S6.** The original gel mobility shift assay of Figure 4 in the main text.
